# Stress Spillover Among Mother-Adolescent Dyads in Mexican Immigrant Families: How It Varies from Early to Late Adolescence

**DOI:** 10.1007/s10964-025-02197-6

**Published:** 2025-05-24

**Authors:** Wen Wen, Ashley Janyn Galvan, Ka I. Ip, Yang Hou, Shanting Chen, Su Yeong Kim

**Affiliations:** 1 Crown Family School of Social Work, Policy, and Practices, University of Chicago, Chicago, IL USA; 2 Department of Human Development and Family Sciences, The University of Texas at Austin, Austin, TX USA; 3 University of Minnesota Institute of Child Development, Minneapolis, MN USA; 4 College of Medicine, Florida State University, Tallahassee, FL USA; 5 Department of Psychology, University of Florida, Gainesville, FL USA

**Keywords:** Mother-adolescent dyads, Mexican-origin, Sociocultural stress, Internalizing symptoms, Time-varying effect

## Abstract

Mothers and adolescent children in Mexican immigrant families may encounter various sociocultural stressors, which may spill over into family interactions and impede each other’s internalizing symptoms based on the Family Systems Theory. Empirical evidence is needed to identify the sensitive developmental age when mothers and adolescents are most vulnerable to each other’s stressors, addressing gaps in understanding *which* types of sociocultural stress can spill over and *when* these spillover effects peak during adolescence. This study adopted a five-year, three-wave dataset that included 604 adolescents (*M*_*Wave1_age*_*(SD)* = 12.92(0.92); 54% female) and 595 mothers (*M*_*Wave1_age*_*(SD)* = 38.89(5.74)) among Mexican immigrant families. Time-varying-effect models showed that the association between maternal stress experiences (cultural misfit, foreigner stress, difficulty paying bills) and adolescent internalizing symptoms was weak to nonsignificant during most of the adolescence period, yet adolescents’ sociocultural stress more strongly influenced maternal symptoms, particularly in early adolescence. These findings underscore the profound impact of adolescents’ sociocultural stress on maternal mental health and highlight the need to consider youth developmental timing when examining such impact.

## Introduction

Immigrants, often perceived as societal outgroups, face mental health challenges due to sociocultural stress such as economic difficulty and discrimination (Huynh, 2012; Roosa et al., 2009; Salas-Wright et al., 2021). The challenges from sociocultural stress can be particularly profound for Mexican immigrant families in the U.S where parents have limited English proficiency and rely on their adolescents to navigate cultural and linguistic barriers. While Family Systems Theory emphasizes familial interconnectedness (Broderick, 1993; Cox & Paley, 1997), prior research predominantly examines the unidirectional transmission from parental – particularly maternal, as mothers are typically the primary caregivers in families (Pakaluk & Price, 2020) – stress to children’s internalizing symptoms. This approach overlooks adolescents’ agency in shaping maternal outcomes. More importantly, although adolescence is widely recognized as a sensitive developmental period influenced by contextual factors (Blakemore & Mills, 2014; Mendoza et al., 2017), there have been limited efforts to identify vulnerable ages within this period relating to stress spillover effects among mother-adolescent dyads. The current study addresses these gaps by investigating how mothers’ and adolescents’ sociocultural stress experiences can be transmitted to influence each other’s internalizing symptoms and how such influences may vary from early to late adolescence among Mexican immigrant families.

### Theoretical Frameworks

Family Systems Theory (Bowen, 1993) and the Stress Spillover phenomenon (Repetti & Bai, 2025) provide complementary lenses to examine how sociocultural stress experienced by mothers and adolescents reciprocally influence each other’s internalizing symptoms (e.g., anxiety, depressive symptoms) and how these dynamics vary across adolescence. Family Systems Theory (Bowen, 1993) posits that mothers and adolescents form an interdependent emotional unit, where sociocultural stress (e.g., cultural misfit, economic stress, and foreigner stress) in one member reverberates through relational dynamics, shaping the other’s internalizing symptoms (e.g., anxiety and depressive symptoms). This interdependence reflects circular causality: a mother’s stress may disrupt parenting practices, exacerbating her adolescent’s distress, while the adolescent’s stress may heighten family tension, worsening maternal mental health. These effects are further explained by the concept of Stress Spillover (Repetti & Bai, 2025), which emphasizes how stress from external contexts (e.g., sociocultural stress experienced by family members) “spill over” into family interactions, disrupting relational harmony and thus impacting members’ mental health.

Family Systems Theory underscores that stress transmission in family dynamics is not static but evolves as adolescents develop autonomy and renegotiate their roles (Olson & Lavee, 2013). Yet gaps remain in understanding how stress spillover among mother-adolescent dyads varies across time. For example, in early adolescence, mothers may be particularly vulnerable to internalizing their adolescent’s distress, as young teens may depend heavily on their mothers’ support due to their limited autonomy. On the other hand, by mid-to-late adolescence, youth’s expanding autonomy and exposure to sociocultural stress further drive the stress spillover, where their stress has a strong impact on maternal internalizing symptoms. In summary, despite theoretical support, empirical ambiguity remains regarding when one family member’s stress most strongly impacts the other’s internalizing symptoms and whether this impact varies depending on the type of stress.

### Maternal Sociocultural Stress and Adolescents’ Internalizing Symptoms

Mothers experiencing high levels of sociocultural stress may inadvertently communicate anxiety or negativity to their adolescent children, who may then internalize these feelings, leading to heightened internalizing symptoms in adolescents such as anxiety and depressive symptoms. Parental cultural stress (e.g., discrimination and negative context of perception) among ethnic minority populations have been shown to contribute to children’s internalizing symptoms (Ford et al., 2013; Lorenzo-Blanco et al., 2017). For example, a longitudinal study conducted on 344 adolescents (ages 14–16) of Mexican descent found that adolescents reported more internalizing symptoms when their parents experienced discrimination and communicated cultural stress to them (Espinoza et al., 2016). Also, a study based on 161 primary caregivers of Mexican-origin girls found that parental cultural stress from racism, acculturation, and political hostility was significantly associated with youth anxiety symptoms (Mullins et al., 2024) However, limited studies have considered cultural stress experiences that occur frequently but are less explicit, such as stress experiences of being perceived as a foreigner and feeling like a misfit. Cultural misfit and foreigner stress may be particularly salient for immigrant-origin individuals in the US who are also ethnic minorities, especially for those who may have darker skin tones or an accent when speaking English (Zou & Cheryan, 2017).

Unlike explicit discrimination, which can be overt and easily identifiable—such as verbal abuse, unequal treatment, or denial of opportunities—foreigner stress and the feeling of being a cultural misfit are often more nuanced and subtle (Huynh, 2012; Pérez Huber & Solorzano, 2015; Sue et al., 2007). These forms of stress manifest in less obvious ways, such as microaggressions or social exclusion. The perception of having foreign characteristics, whether it is due to language, accent, or physical appearance, can lead to a constant awareness of being different in various social settings. For mothers in Mexican-origin immigrant families, these experiences can create a persistent sense of not belonging, which can be transmitted to their adolescent children during their interactions and communications (Bowers & Yehuda, 2016). Adolescents, at a crucial period of identity development and driven by a desire for social belonging and acceptance, may be particularly sensitive to maternal cues of misfit or foreignness, potentially leading to increased internalizing symptoms. Such sensitivity may be more salient in early adolescence when children start to develop their sense of ethnic identity and establish peer relationships (Sebastian et al., 2010; Somerville, 2013).

Other than the stress derived from feelings of being a misfit or foreigner, financial difficulties may also create significant stress for Mexican immigrant families (Noe-Bustamante et al., 2019). Because of structural racism (e.g., racial residential segregation and lack of intergenerational wealth), some Mexican immigrant families may face significant barriers to accessing socioeconomic opportunities. These challenges include fewer resources for pursuing higher education and finding high-paying jobs (Flores & Chapa, 2008), particularly for immigrant parents with limited English skills. Indeed, 14% of Mexican families in the United States reported experiencing bill-paying hardship (a proxy of economic hardship), compared to only 9% of non-Hispanics (Scherer & Mayol-Garcia, 2022). Such economic pressure experienced by parents can be transmitted and lead to more internalizing symptoms in adolescents (Kavanaugh et al., 2018; Neppl et al., 2016). However, it is unknown whether maternal economic pressure may have different associations with adolescents’ internalizing symptoms at different ages, from early to late adolescence. As adolescents develop, they may acquire a better understanding of their mother’s financial/emotional situation (i.e., understand the mother’s stress) compared to when they were younger. Evidently, adolescents develop empathy and perspective-taking abilities as they mature from early to late adolescence (Larson et al., 2002; Smith & Carlson, 1997). Therefore, we test for the possibility that older adolescents may be more responsive to maternal stress experiences and show greater internalizing symptoms.

### Adolescents’ Sociocultural Stress Experiences and Maternal Internalizing Symptoms

Adolescents’ experiences of sociocultural stress can be a significant concern for their mothers (Harcourt et al., 2014), who often serve as primary caregivers and are expected to provide emotional support. Given the critical role that mothers play in addressing adolescents’ emotional needs, adolescents’ perceived stress may, in turn, be transmitted to their mothers, potentially contributing to elevated levels of maternal internalizing symptoms. For example, parents who have adolescent children experiencing more (vs. low) discrimination report less positive affect among a Latinx sample in the US (Bámaca et al., 2022).

Although only a few empirical studies have examined such an association in terms of ethnic minority adolescents’ sociocultural stress experiences and mothers’ internalizing symptoms (Bámaca et al., 2022), some studies have also provided preliminary evidence of such an association occurring with other stress experiences (Harcourt et al., 2014; Nomaguchi & Fettro, 2020; Sawyer et al., 2011). For example, a previous study found that for children who experience more victimization/bullying, a type of stress experience, their mothers reported more depressive symptoms, perhaps due to feeling responsible for the bullying of their children (Nomaguchi & Fettro, 2020). A few studies have considered the developmental period of adolescence in terms of how stress experienced by adolescents may influence their mothers. Given the great changes in cognition and physical traits that they experience, adolescents may play a critical role in driving the stress response of their mothers. For example, early adolescence is a crucial developmental period because it marks the transition from childhood to adolescence, so mothers tend to worry more about their children’s stress experiences during this period because of concerns about the challenges that occur during transitions (Larson et al., 2002, Forbes & Dahl 2010). In other words, the association between child stress experiences and maternal internalizing symptoms may be stronger in early adolescence than in middle or late adolescence. Examining the potential influence of adolescents’ stress on maternal internalizing symptoms may be particularly salient in immigrant families with language broker adolescents who play an important role in helping mothers adapt to mainstream society (Kam & Lazarevic, 2014).

## The Current Study

Prior research has established that maternal or adolescents’ sociocultural stress can exacerbate each other’s internalizing symptoms, yet gaps remain in identifying the critical developmental periods when these dyads are most vulnerable to each other’s sociocultural stress and whether such influence varies across different types of sociocultural stress. The current study aimed to illustrate how adolescents’ and mothers’ sociocultural stress experiences may be related to the other’s internalizing symptoms (i.e., anxiety and depressive symptoms) and how such associations may vary over time. Two hypotheses were made based on the Family Systems Theory. First, maternal cultural stress related to being foreigners and misfits would be related to higher adolescents’ internalizing symptoms, and this association would be stronger in early versus late adolescence. On the other hand, maternal economic stress would be related to adolescents’ higher internalizing symptoms in late but not early adolescence. Second, adolescents’ sociocultural stress would be related to higher maternal internalizing symptoms, and this association would be more profound in early but not late adolescence. The study was not preregistered.

## Methods

### Participants

The current study collected longitudinal data from adolescents and mothers from Mexican immigrant families in three waves across approximately 5 years (Kim et al., 2024). The three waves sampled 604 adolescents (54% female) and 595 mothers living in and around central Texas in the United States. At Wave 1 (2012–2015) adolescent participants were 11–15 years old (*M* = 12.92, *SD* = 0.92) in middle school (6th–8th grade) and mothers were 27–65 years old (*M* = 38.89, *SD* = 5.74). Most of the adolescent participants lived with both of their parents (76%) and were born in the United States (75%), and the median family income was between $20,001 and $30,000. Most youth (91.7%) qualified for free or reduced-price lunch at school. The majority of youth (86.3%) reported mothers as the primary caregiver. Almost all mothers were born in Mexico (99%), and the average age of arriving in the U.S. to live permanently was 23.31. At Wave 2 (1 year later), 80% of adolescents (*n* = 483, 55% female; *M*_age_ = 13.72, *SD* = 0.90) and 81% of mothers (*n* = 479, *M*_age_ = 39.80, *SD* = 5.85) continued participating in the study. At Wave 3 (4 years after Wave 2), 55% of adolescents (*n* = 334, 56% female; *M*_age_ = 17.62, *SD* = 1.05) and 55% of mothers (*n* = 329, *M*_age_ = 43.85, *SD* = 5.75) continued their participation in the study. All available data from three waves were included in the analysis, including participants who only participated in one or two waves. To investigate the pattern of missing data from families who did not continue their participation across study waves, attrition analysis was performed to identify differences in study variables and demographics (i.e., adolescent age, gender, nativity, and maternal education). Based on the results, families that remained in the study for Wave 2 showed higher maternal education levels, *t* (591) = 2.41, *p* < 0.05. Families that remained in the study from Wave 2 to Wave 3 were found to have younger adolescent participants, *t* (481) = 2.97, *p* < 0.01.

### Procedure

Participants were recruited between 2012 and 2015 through public records, school presentations, and community recruitment in and around a large metropolitan city in central Texas in the United States. Eligibility was met if the families were of Mexican origin and had at least one child who translated for their parents at minimum once every 6 months. The recruitment process and eligibility screening were conducted by undergraduate research assistants with the supervision of a senior research assistant and the principal investigator of the larger study. Once families were deemed qualified for the project, bilingual undergraduate research assistants scheduled an acquaintance meeting with the family to obtain personal information, provide them with information about the project and their compensation, and obtain verbal consent (from parents) and assent (from adolescents).

After the acquaintance visit, bilingual undergraduate research assistants would schedule formal home interviews with the families, in which they would administer a long survey in the participants’ preferred language (English or Spanish). The English items were initially translated into Spanish and then back-translated into English by bilinguals to ensure the accuracy and validity of the translation. During this visit, participants verbally answered survey questions related to their economic hardship experiences, cultural misfit experiences, foreigner stress, and internalizing symptoms. Verbal answers were recorded on a laptop computer by bilingual and bicultural interviewers. Data for all waves were collected with the same procedure. Wave 2 was conducted 1 year after Wave 1, and Wave 3 was conducted 4 years after Wave 2. Each family received compensation of $60 for Wave 1, and $90 for Wave 2 and Wave 3.

### Measures

#### Perceived Economic Stress

For perceived economic stress, adolescents self-reported, “How much of a problem did your family have because your parents did not have enough money to buy things your family needs or wants?” (Mistry et al., 2009), and mothers self-reported, “How much financial difficulty did you have with paying your bills?” based on their experiences in the past 3 months on a scale ranging from 1 (*not at all*) to 5 (*very*). Higher mean scores reflect experiencing more economic stress.

#### Foreigner Stress

Foreigner stress was measured with a four-item scale developed and validated in a previous study (Kim et al., 2018). Adolescents and mothers self-reported how much they agreed with statements such as, “When people look at me, they see a foreigner,” on a scale ranging from 1 (*strongly disagree*) to 5 (*strongly agree*). Higher mean scores reflect having more experiences of being perceived as a foreigner (adolescents: α_wave 1_ = 0.711, α _wave 2_ = 0.757, α _wave 3_ = 0.781; mothers: α _wave 1_ = 0.689, α _wave 2_ = 0.699, α _wave 3_ = 0.739). Mother and adolescent reported foreigner stress met scalar and partial scalar longitudinal measurement invariance, respectively (Table S1).

#### Cultural Misfit

Cultural misfit was measured by a four-item scale developed through the Cultural Estrangement Inventory (Cozzarelli & Karafa, 1998). Adolescents and mothers self-reported how much they agreed with statements such as, “I feel as though most U.S. Americans do not understand me,” on a scale ranging from 1 (*strongly disagree*) to 5 (*strongly agree*). Higher average scores reflect higher levels of feeling like a cultural misfit (adolescents: α _wave 1_ = 0.773, α _wave 2_ = 0.772, α _wave 3_ = 0.769; mothers: α _wave 1_ = 0.731, α _wave 2_ = 0.758, α _wave 3_ = 0.723). Both mother and adolescent reported cultural misfit met scalar longitudinal measurement invariance (Table S2).

#### Depressive Symptoms

Depressive symptoms were assessed using the 20-item Center for Epidemiologic Studies Scale (Radloff, 1977). A sample item is, “I was bothered by things that I am usually not bothered by”. Adolescents and mothers self-reported their experiences in the past week on a scale ranging from 0 (*rarely or none*) to 3 (*most or all of the time*). Higher average scores reflect higher levels of depressive symptoms (adolescents: α _wave 1_ = 0.833, α _wave 2_ = 0.844, α _wave 3_ = 0.866; mothers: α _wave 1_ = 0.884, α _wave 2_ = 0.887, α _wave 3_ = 0.847). Both mother and adolescent reported depressive symptoms met scalar longitudinal measurement invariance (Table S3).

#### Anxiety Symptoms

Anxiety symptoms were assessed using four items from previous studies (Reynolds & Richmond, 1978; Spitzer et al., 2006): Adolescents and mothers self-reported their experiences in the past 2 weeks on a scale ranging from 1 (*rarely or none*) to 4 (*nearly every day*). Higher average scores reflect higher levels of anxiety symptoms (adolescents: α _wave 1_ = 0.750, α _wave 2_ = 0.822, α _wave 3_ = 0.711; mothers: α _wave 1_ = 0.809, α _wave 2_ = 0.842, α _wave 3_ = 0.816). Both mother and adolescent reported foreigner stress met scalar longitudinal measurement invariance (Table S4).

### Analysis Plan

Analysis was conducted in two steps. First, descriptive information and correlation of study variables were conducted in R 4.1.1. Second, 12 time-varying effect models (i.e., TVEM) were estimated to examine how three types of sociocultural stress influence two types of internalizing symptoms for mothers and adolescents. Mothers’ and adolescents’ perceptions of sociocultural stress were modeled together in the same model to control for each other’s experiences. TVEM is a type of nonparametric estimation examining continuous changes in the associations between variables across time. We conducted TVEM in SAS 9.4 using the %TVEM program with the 95% confidence interval (i.e., CI) estimated. In the current analysis, P-spline functions with 10 knots were chosen to provide a robust and automatic estimation of the association. The decision to use 10 knots was based on a balance between model complexity and capturing the nuances of the time-varying effect (Lanza & Linden-Carmichael, 2021).

The analysis followed a two-step procedure to interpret the findings. The first was to determine whether there was a significant association between sociocultural stress and internalizing symptoms by checking whether the confidence intervals overlap (i.e., non-significant) or exclude (i.e., significant) the line of zero at the examined ages. The second step was to determine whether there was a significant change in the association by examining whether the confidence intervals overlap. It would be considered a significant change when the highest point of the lower confidence interval of the coefficient curve was larger than the lowest point of the higher confidence interval at any age within the examined range. The increase and the decrease of the association depended on the overall trend of the association over time. The data were structured in a long format, with the analysis unit being each wave for each mother-adolescent dyad (i.e., waves nested within mother-adolescent dyads). Adolescent age was the time variable in the models. An example equation is:

$${\widehat{{y}_{ij}}}={\beta }_{0}({{age}}_{{ij}})+{\beta }_{1}({{age}}_{{ij}}){{mother\; stress}}_{{ij}}+{\beta }_{2}({{age}}_{{ij}}){{adolescent\; stress}}_{{ij}}+{\beta }_{3}\,{{time\; invariant\; covaraites}}_{i}+{\varepsilon }_{{ij}}.$$

${{Mother\; stress}}_{{ij}}$ and ${{adolescent\; stress}}_{{ij}}$ represents the stress experienced for mother-adolescent dyad i at time j; ${\beta }_{1}{({age}}_{{ij}})$ represents the time-varying coefficient for the association between stress experiences and the outcome (i.e., depressive symptoms or anxiety); ${\beta }_{0}{({age}}_{{ij}})$ and ${\varepsilon }_{{ij}}$ represents the time-varying intercept and error across adolescents’ age, respectively. The analysis does not distinguish within- versus between-person effects but focuses on the change of associations between sociocultural stress and internalizing symptoms among mother-adolescent dyads across time. Figure S1 presents the distribution of adolescent ages for all waves. Time-invariant covariates that may be related to internalizing symptoms were controlled in the models. Specifically, maternal education level, adolescent gender, and adolescent nativity were included in all models. The syntax for the time-varying effect model analysis is available in OSF repository, which can be accessed at https://osf.io/xkfb6/?view_only=5eee15951d6c4d71a9be45436b72f611 (Wen, 2024). Additionally, maternal age was included as a covariate for models with maternal internalizing symptoms. Sensitivity analyses with a 99% confidence interval were conducted to test the robustness of the results and to reduce the potential for an inflated Type 1 error when testing multiple models with a 95% confidence interval.

## Results

The descriptive information and correlation of study variables are shown in Table 1. For the time-varying effect models, although the full age range represented in the sample is presented in all figures, only estimates provided for ages 11.5–19 are interpreted. Estimates for children younger than 11.5 (*n* = 26) or older than 19 (*n* = 28) are not interpreted because of the wide CI due to the limited sample size in those age ranges. This study described early, middle, and late adolescence as ages 11.5–13.9, ages 14–17.9, and ages 18–19, respectively (Steinberg, 2019).

**Table 1:** Descriptive information and correlation of study variables

Variable	*M*	*SD*	1	2	3	4	5	6	7
1. Adolescent female W1	0.543	0.499	—						
2. Adolescent foreign-born W1	0.247	0.431	−0.022	—					
3. Mother education W1	4.811	2.201	0.001	0.021	—				
4. Adolescent cultural misfit W1	2.309	0.674	−0.075	0.083^*^	−0.037	—			
5. Adolescent foreigner stress W1	2.426	0.731	−0.064	0.089^*^	−0.062	0.577^***^	—		
6. Adolescent difficulty paying bills W1	3.998	0.940	0.049	0.067	−0.040	−0.199^***^	−0.162^***^	—	
7. Mother cultural misfit W1	2.643	0.775	−0.020	0.069	−0.062	0.021	0.079	−0.013	—
8. Mother foreigner stress W1	3.467	0.800	0.011	0.027	0.051	0.039	0.016	−0.067	0.346^***^
9. Mother difficulty paying bills W1	2.269	0.982	0.070	0.018	0.014	0.032	−0.014	−0.141^***^	0.041
10. Adolescent depressive symptoms W1	1.562	0.385	0.083^*^	0.071	0.053	0.279^***^	0.198^***^	−0.268^***^	0.069
11. Adolescent anxiety W1	1.692	0.613	0.101^*^	0.023	0.055	0.275^***^	0.223^***^	−0.240^***^	0.028
12. Mother depressive symptoms W1	1.470	0.423	0.038	−0.022	−0.074	0.067	0.097^*^	−0.041	0.162^***^
13. Mother anxiety W1	1.706	0.660	0.037	0.009	−0.079	0.097^*^	0.052	−0.125^**^	0.197^***^
14. Adolescent cultural misfit W2	2.239	0.652	0.007	0.023	−0.033	0.417^***^	0.358^***^	−0.113^*^	0.040
15. Adolescent foreigner stress W2	2.418	0.729	−0.029	0.074	−0.044	0.356^***^	0.508^***^	−0.075	0.062
16. Adolescent difficulty paying bills W2	4.206	0.851	−0.034	0.003	−0.056	−0.220^***^	−0.210^***^	0.348^***^	−0.027
17. Mother cultural misfit W2	2.507	0.772	−0.064	0.059	−0.071	0.089	0.126^**^	0.070	0.423^***^
18. Mother foreigner stress W2	3.376	0.800	−0.046	0.030	0.063	0.007	0.046	0.013	0.227^***^
19. Mother difficulty paying bills W2	2.134	0.894	0.046	−0.020	−0.050	0.102^*^	−0.023	−0.104^*^	0.063
20. Adolescent depressive symptoms W2	1.547	0.388	0.187^***^	0.092^*^	0.048	0.194^***^	0.134^**^	−0.085	0.004
21. Adolescent anxiety W2	1.720	0.651	0.192^***^	0.105^*^	0.093^*^	0.150^***^	0.143^**^	−0.097^*^	0.019
22. Mother depressive symptoms W2	1.473	0.421	−0.032	−0.014	−0.091^*^	0.084	−0.000	−0.042	0.134^**^
23. Mother anxiety W2	1.690	0.686	0.009	−0.031	−0.038	0.065	0.003	−0.068	0.145^**^
24. Adolescent cultural misfit W3	2.422	0.660	−0.008	0.093	0.035	0.283^***^	0.246^***^	−0.103	0.022
25. Adolescent foreigner stress W3	2.448	0.742	−0.028	0.079	0.027	0.172^**^	0.345^***^	0.028	0.079
26. Adolescent difficulty paying bills W3	4.255	0.856	−0.007	−0.045	−0.066	−0.187^***^	−0.209^***^	0.285^***^	−0.023
27. Mother cultural misfit W3	2.502	0.706	0.021	−0.019	−0.062	0.051	0.047	−0.030	0.361^***^
28. Mother foreigner stress W3	3.333	0.834	0.131^*^	0.001	0.099	−0.068	−0.047	0.010	0.159^**^
29. Mother difficulty paying bills W3	2.003	0.850	−0.011	−0.036	−0.033	0.051	0.020	−0.099	0.117^*^
30. Adolescent depressive symptoms W3	1.545	0.376	0.076	0.010	0.098	0.203^***^	0.145^**^	−0.064	0.016
31. Adolescent anxiety W3	1.847	0.672	0.166^**^	0.057	0.087	0.190^***^	0.144^**^	−0.077	0.047
32. Mother depressive symptoms W3	1.464	0.370	−0.082	0.019	−0.073	0.137^*^	0.086	−0.059	0.167^**^
33. Mother anxiety W3	1.626	0.638	0.064	0.016	−0.016	0.149^**^	0.089	−0.036	0.178^**^

Variable	8	9	10	11	12	13	14	15	16	17	18	19	20
9. Mother difficulty paying bills W1	0.108^**^	—											
10. Adolescent depressive symptoms W1	0.021	0.077	—										
11. Adolescent anxiety W1	0.003	0.084^*^	0.610^***^	—									
12. Mother depressive symptoms W1	0.112^**^	0.175^***^	0.082^*^	0.106^**^	—								
13. Mother anxiety W1	0.128^**^	0.294^***^	0.109^**^	0.104^*^	0.574^***^	—							
14. Adolescent cultural misfit W2	0.085	0.070	0.211^***^	0.169^***^	0.086	0.094^*^	—						
15. Adolescent foreigner stress W2	0.034	0.031	0.161^***^	0.125^**^	0.090^*^	0.104^*^	0.570^***^	—					
16. Adolescent difficulty paying bills W2	−0.067	−0.159^***^	−0.245^***^	−0.258^***^	−0.035	−0.098^*^	−0.205^***^	−0.171^***^	—				
17. Mother cultural misfit W2	0.239^***^	0.072	−0.010	−0.039	0.203^***^	0.223^***^	0.103^*^	0.109^*^	0.039	—			
18. Mother foreigner stress W2	0.417^***^	0.117^*^	0.050	0.033	0.078	0.142^**^	0.087	0.043	−0.026	0.436^***^	—		
19. Mother difficulty paying bills W2	0.087	0.428^***^	0.100^*^	0.030	0.142^**^	0.251^***^	0.050	0.006	−0.210^***^	0.092^*^	−0.000	—	
20. Adolescent depressive symptoms W2	0.005	0.052	0.529^***^	0.404^***^	0.117^*^	0.112^*^	0.231^***^	0.167^***^	−0.249^***^	−0.025	0.019	0.073	—
21. Adolescent anxiety W2	−0.016	0.033	0.410^***^	0.507^***^	0.128^**^	0.114^*^	0.195^***^	0.146^**^	−0.241^***^	0.000	0.019	0.009	0.625^***^
22. Mother depressive symptoms W2	0.052	0.237^***^	0.040	0.009	0.504^***^	0.414^***^	0.086	0.077	−0.102^*^	0.256^***^	0.110^*^	0.168^***^	0.102^*^
23. Mother anxiety W2	0.138^**^	0.257^***^	0.066	0.032	0.429^***^	0.566^***^	0.060	0.060	−0.118^*^	0.202^***^	0.172^***^	0.260^***^	0.105^*^
24. Adolescent cultural misfit W3	0.074	0.043	0.182^***^	0.172^**^	−0.053	−0.029	0.315^***^	0.265^***^	−0.148^**^	0.134^*^	0.051	−0.049	0.152^**^
25. Adolescent foreigner stress W3	0.014	0.028	0.066	0.108^*^	0.018	0.027	0.179^**^	0.399^***^	−0.096	0.102	−0.017	−0.024	0.130^*^
26. Adolescent difficulty paying bills W3	−0.021	−0.188^***^	−0.161^**^	−0.133^*^	−0.018	−0.081	−0.139^*^	−0.171^**^	0.446^***^	−0.059	−0.021	−0.136^*^	−0.157^**^
27. Mother cultural misfit W3	0.237^***^	0.089	0.084	−0.007	0.116^*^	0.149^**^	0.104	0.096	−0.005	0.344^***^	0.224^***^	0.088	−0.055
28. Mother foreigner stress W3	0.378^***^	0.115^*^	0.096	0.002	0.038	0.088	0.042	0.057	−0.019	0.209^***^	0.362^***^	0.036	0.028
29. Mother difficulty paying bills W3	−0.002	0.316^***^	0.071	0.011	0.085	0.151^**^	0.088	0.104	−0.161^**^	0.032	0.078	0.403^***^	0.035
30. Adolescent depressive symptoms W3	0.036	0.002	0.271^***^	0.304^***^	−0.055	−0.015	0.223^***^	0.251^***^	−0.179^**^	0.035	−0.002	−0.034	0.352^***^
31. Adolescent anxiety W3	0.046	0.055	0.253^***^	0.315^***^	−0.041	0.008	0.192^***^	0.208^***^	−0.217^***^	−0.008	−0.004	−0.007	0.326^***^
32. Mother depressive symptoms W3	0.078	0.044	0.059	0.005	0.359^***^	0.346^***^	0.152^**^	0.079	−0.049	0.276^***^	0.140^*^	0.110	0.034
33. Mother anxiety W3	0.116^*^	0.149^**^	0.009	−0.041	0.364^***^	0.520^***^	0.135^*^	0.052	−0.113^*^	0.256^***^	0.177^**^	0.209^***^	0.057

Variable	21	22	23	24	25	26	27	28	29	30	31	32
22. Mother depressive symptoms W2	0.060	—										
23. Mother anxiety W2	0.081	0.539^***^	—									
24. Adolescent cultural misfit W3	0.205^***^	−0.043	−0.041	—								
25. Adolescent foreigner stress W3	0.174^**^	−0.068	−0.026	0.406^***^	—							
26. Adolescent difficulty paying bills W3	−0.193^***^	−0.085	−0.104	−0.247^***^	−0.198^***^	—						
27. Mother cultural misfit W3	−0.058	0.190^***^	0.146^*^	0.119^*^	−0.018	−0.074	—					
28. Mother foreigner stress W3	0.011	0.028	0.063	0.072	−0.022	−0.070	0.457^***^	—				
29. Mother difficulty paying bills W3	−0.018	0.136^*^	0.131^*^	0.084	0.019	−0.181^**^	0.078	0.043	—			
30. Adolescent depressive symptoms W3	0.303^***^	−0.026	−0.001	0.378^***^	0.239^***^	−0.294^***^	0.086	0.058	0.010	—		
31. Adolescent anxiety W3	0.388^***^	−0.033	−0.101	0.379^***^	0.172^**^	−0.310^***^	0.033	0.025	0.115^*^	0.676^***^	—	
32. Mother depressive symptoms W3	−0.050	0.336^***^	0.261^***^	0.016	−0.022	−0.004	0.235^***^	0.091	0.116^*^	0.022	−0.004	—
33. Mother anxiety W3	−0.006	0.338^***^	0.453^***^	0.002	0.008	−0.083	0.183^***^	0.084	0.206^***^	0.042	0.066	0.511^***^

*p < 0.05. **p < 0.01. ***p < 0.001

### Association of Maternal Stress Experiences with Adolescent Internalizing Symptoms

**Fig. 1:**
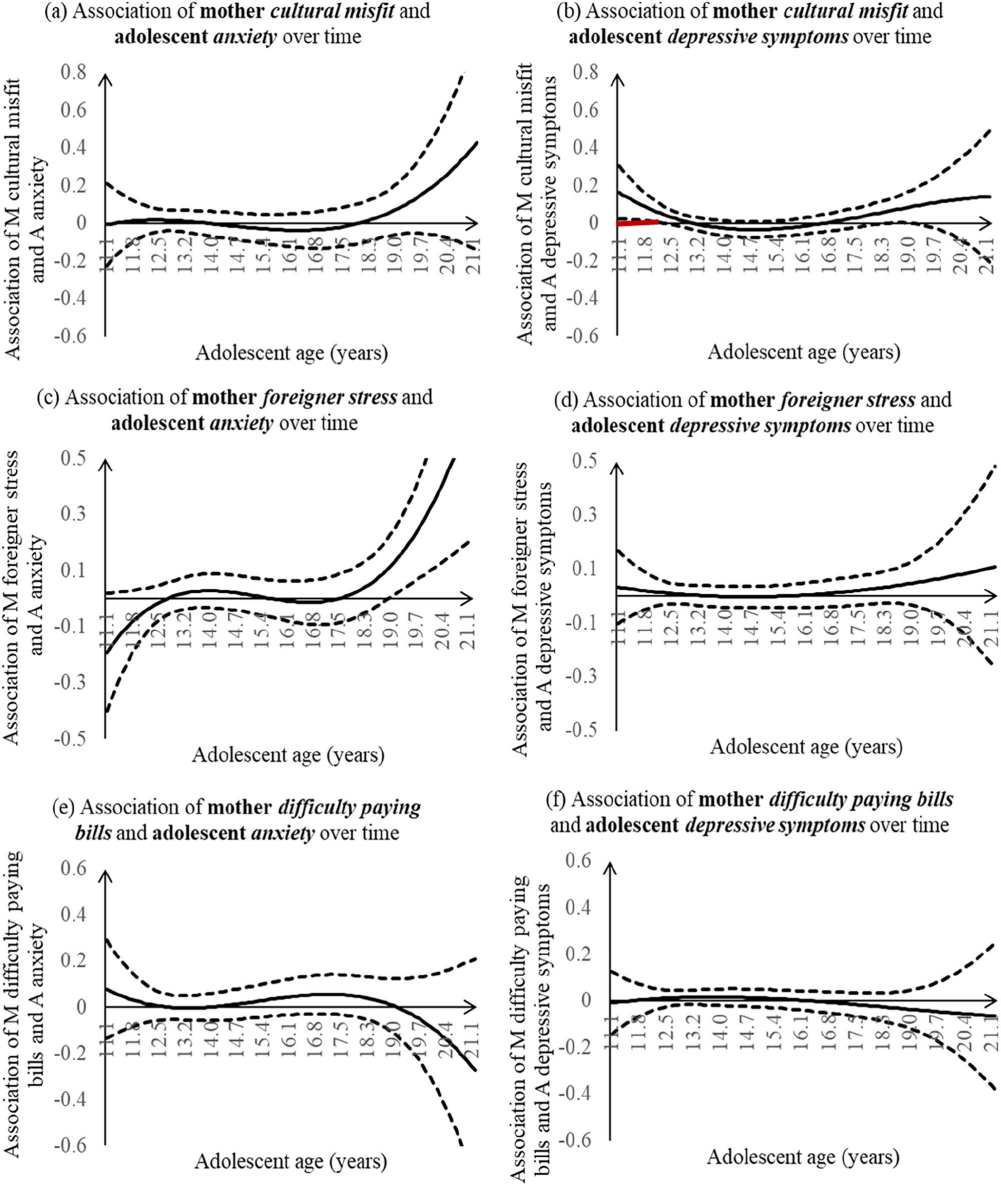
Associations of maternal sociocultural stress and youth internalizing symptoms across time. Dashed lines represent point wise 95% confidence intervals. Age ranges with significant associations were highlighted in bold red lines. A adolescent, M mother

Maternal cultural misfit (Fig. 1a & b) was associated with more adolescent depressive symptoms from age 11.5 (b = 0.116, 95% CI [0.017, 0.215]) to age 12.3 (b = 0.044, 95% CI [0.000, 0.089]) but not to adolescent anxiety at any age. The associations between mothers’ cultural misfit and adolescents’ internalizing symptoms did not change during the period when there was a significant association (i.e., age 11.5–12.3 for depressive symptoms). Maternal foreigner stress (Fig. 1c & d) or difficulty paying bills (Fig. 1e & f) was not related to adolescent anxiety or depressive symptoms within the explained age range.

### Association of Adolescent Stress Experiences with Maternal Internalizing Symptoms

**Fig. 2:**
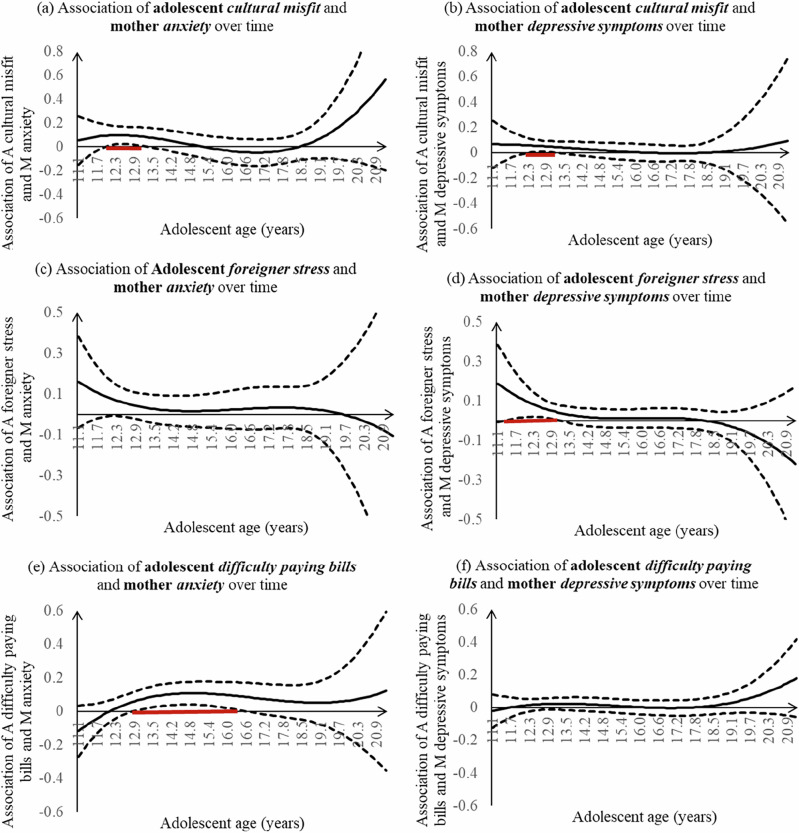
Associations of youth sociocultural stress and maternal internalizing symptoms across time. Dashed lines represent point wise 95% confidence intervals. Age ranges with significant associations were highlighted in bold red lines. A adolescent, M mother

In general, there were positive associations between adolescent cultural misfit, foreigner stress, difficulty paying bills, and maternal internalizing symptoms, mainly in early adolescence. Adolescent cultural misfit (Fig. 2a) was associated with more maternal anxiety from adolescent age 12.0 (b = 0.093, 95% CI [0.003, 0.184]) to age 13.3 (b = 0.081, 95% CI [0.002, 0.160]). Adolescents’ experiences of cultural misfit were associated with more depressive symptoms for their mothers between adolescent age 12.3 (b = 0.059, 95% CI [0.001, 0.116]) to age 13.2 (b = 0.046, 95% CI [0.001, 0.091] (Fig. 2b). Adolescent foreigner stress (Fig. 2c & d) was not related to higher levels of maternal anxiety, but it was related to more maternal depressive symptoms from adolescent age 11.5 (b = 0.146, 95% CI [0.007, 0.284]) to age 13.1 (b = 0.042, 95% CI [0.003, 0.080]). Adolescent-reported difficulty paying bills (Fig. 2e & f) was positively associated with maternal anxiety from adolescent age 12.9 (b = 0.059, 95% CI [0.002, 0.117]) to age 16.5 (b = 0.087, 95% CI [0.003, 0.171]) but was not associated with maternal depressive symptoms. The associations between adolescents’ stress experiences and their mothers’ internalizing symptoms did not vary across adolescent ages.

### Sensitivity Analysis Results

As multiple models were tested in the current study, more conservative analyses were conducted (i.e., sensitivity analyses with a 99% confidence interval) to test the robustness of the results after considering the possibility of inflated Type 1 error due to multiple tests. Sensitivity analysis results are presented in supplementary figures S2–S3. Different from the main results showing the positive association between maternal cultural misfit and adolescent depressive symptoms in early adolescence, there was no significant association between maternal perceived sociocultural stress and adolescents’ internalizing symptoms with the 99% confidence interval. Consistent with the main results, sensitivity results reveal similar patterns of association between adolescents’ perceived sociocultural stress and mothers’ internalizing symptoms. More adolescent cultural stress was associated with more maternal internalizing symptoms in their children’s early adolescence (i.e., adolescents’ cultural misfit and foreigner stress to mothers’ anxiety and depressive symptoms, respectively); higher levels of adolescent-perceived difficulty paying bills were related to more maternal anxiety in middle adolescence.

## Discussion

While maternal and adolescent sociocultural stress has been shown to impede each other’s internalizing symptoms, gaps remain in identifying the developmental periods of greatest vulnerability for this influence and revealing whether this influence varies by type of sociocultural stress. The current study built on Family Systems Theory (Broderick, 1993; Cox & Paley, 1997) and takes a developmental perspective to understand how adolescents’ and mothers’ sociocultural stress experiences may be related to each other’s internalizing symptoms (i.e., anxiety and depressive symptoms) and how such associations may vary over time. The findings reveal a spillover effect of adolescents’ cultural stress of being perceived as foreigners or misfits on maternal internalizing symptoms in early adolescence, as well as a spillover effect of adolescents’ economic stress experiences on maternal anxiety in middle adolescence. However, inconsistent with previous studies and hypotheses, the associations between maternal stress experiences and adolescent internalizing symptoms were relatively weak to nonsignificant during most of the adolescent period. This study revealed crucial time windows when mothers are vulnerable to youth sociocultural stress experience (i.e., early adolescence for cultural stress and middle adolescence for economic stress) and highlighted the need to study sociocultural stress experiences within the family systems.

### The Association of Maternal Sociocultural Stress and Adolescent Internalizing Symptoms

Inconsistent with the hypothesis, most associations between maternal sociocultural stress and adolescent internalizing symptoms were not significant at any age, suggesting that such associations, if any, may not be as strong in the current sample as those found in previous studies (e.g., Bowers & Yehuda, 2016). The different findings may be partly due to the focus on different sociocultural stress experiences. Unlike explicit discrimination, mothers may tend to internalize their feelings related to being perceived as foreigners or misfits and are less likely to communicate those experiences with children, as shown by the lack of variations in children’s internalizing symptoms along with maternal cultural stress experiences. Also, families of the current sample are in general economically disadvantaged because of multiple systematic barriers to accessing well-paying jobs (Hong et al., 2022). Adolescents in such families may be well aware of the economic stress that the family is experiencing. Thus, maternal perceived economic stress may have little influence on youth internalizing symptoms beyond the youth’s own perceptions of economic stress.

In addition, this study captured parents’ and adolescents’ perspectives on their sociocultural experiences and internalizing symptoms, ensuring a more accurate reflection of their lived experiences. Given the well-documented discrepancies between parental and adolescent reports on adolescent internalizing symptoms (e.g, Hughes & Gullone, 2010), studies that rely solely on parental reports may overestimate the link between parental cultural stress and youth internalizing symptoms.(e.g., Mullins et al., 2024). Also, the current study took into consideration the influence of adolescents’ own sociocultural stress experiences when examining the association between maternal sociocultural stress and youth internalizing symptoms, so the non-significant findings are more conservative and robust. Adolescents’ sociocultural stress experiences may be distinct from those that their mothers encounter, as they are often more acculturated and endorse more mainstream cultural values and beliefs than their mothers do (Smokowski et al., 2008), a notion that is supported by the null to small correlations between adolescents’ and mothers’ sociocultural stress experiences. This difference between mothers and adolescents may create a sense of distance (Smokowski et al., 2008), which may contribute to the lack of association between maternal sociocultural stress and adolescent internalizing symptoms.

Maternal cultural misfit feelings were related to higher adolescent depressive symptoms in early adolescence, after accounting for the role of adolescents’ own stress experiences. However, such associations were no longer significant in the sensitivity analyses with the 99% confidence interval, speaking to the need to be extra cautious when interpreting the current findings. It is possible that the significant associations in early adolescence may reflect adolescents’ sensitivity to social information related to their identity development during this period (Forbes & Dahl, 2010). Maternal experiences of being a misfit may be transferred to them as youth share certain marginalized identities (e.g., immigrant-origin and Mexican origin) with their mothers. However, future studies with a larger sample size or more time points and examinations of underlying mechanisms are needed to further test this interpretation.

### The Association of Adolescent Stress and Maternal Internalizing Symptoms

The findings suggest that adolescent cultural misfit and foreigner stress were generally associated with higher levels of maternal anxiety and depressive symptoms during their children’s early adolescence after accounting for mothers’ own stress experiences. The results are consistent with previous studies showing that mothers’ internalizing symptoms may be influenced by their adolescents’ stress experiences (e.g., Nomaguchi & Fettro, 2020). The current study highlighted that the association between adolescents’ sociocultural stress experiences and mothers’ internalizing symptoms may vary by developmental timing. In the current sample, the association between adolescents’ cultural stress and mothers’ internalizing symptoms was only significant during early adolescence. In early adolescence, children experience substantial emotional and physical changes that they start learning to navigate, which may require more attention and guidance from mothers during this period of transition. In addition, early adolescence is a critical age for ethnic identity development and a pivotal developmental window for parental racial socialization (Blakemore & Mills, 2014; Sebastian et al., 2010). As a result, mothers assume a vital role during this period with greater engagement with youth and thus may be more influenced by their adolescents’ stress experiences. On the other hand, as children enter middle adolescence, they become more likely to disclose their stress experiences to peers as opposed to their mothers (Sameroff, 2010), potentially leaving mothers of older adolescents less aware of adolescent stress experiences.

Economic stress, unlike cultural stress, was found by the current study to be related to higher levels of maternal anxiety during middle adolescence, but not during early adolescence. This may be because children tend to develop more financial responsibility, comprehension, and independence during the course of adolescence (Butterbaugh et al., 2020), making adolescents’ perceptions regarding difficulty paying bills a concern for mothers. The legal age of employment is 14 in Texas (Texas Child Labor Law, 2023), which maps onto the significant association between adolescents’ perceived household economic stress and maternal anxiety when children are around age 14. As found in prior research, parents may be generally concerned about their child’s working hours, work safety, or financial responsibility (Beskin & Caskey, 2019; Runyan et al., 2009). Therefore, adolescents’ perceptions of economic stress may be more salient and important for mothers and thus related to greater maternal anxiety only in middle adolescence.

### Limitations and Future Directions

The current study addressed several gaps in the previous literature by providing a greater understanding of how sociocultural stress may be related to internalizing symptoms across the course of adolescence in mother-adolescent dyads in Mexican immigrant families. Despite its strengths, the current study had some limitations. First, this study focused on mother-adolescent dyads only, and the experiences of fathers were not considered. Fathers may play a significant role in family functioning and could have a different influence on adolescent development. In Latinx families, fathers are often the financial providers and thus may have more interactions with the mainstream culture, which could produce its own set of sociocultural stressors. A future study could include fathers’ experiences and consider how associations of stress experiences and internalizing symptoms that emerge in father-adolescent dyads may vary over time.

Another limitation is that only one item was used to measure economic stress for both mothers and adolescents. To address this limitation, future studies can consider different experiences related to economic stress that are salient in the lives of Mexican immigrant families. By measuring multiple aspects of economic stress, future studies will be able to gain a better understanding of the role economic stress plays in the well-being of this low-income population.

It is important to note that early, middle, and late adolescence may be defined differently in research focusing on different domains of development, and interpretations of the current findings need to be made with caution. In addition, the current study structured the longitudinal data length-wise based on participants’ exact age at each wave and analyzed the data cross-sectionally to test the age effect. Despite the strength of this approach in increasing the sample size for each age group, the current study was limited in the sample size after age 15 due to attrition over five years. Future studies collecting more waves of intensive longitudinal data with a larger sample size would be ideal for testing time-varying associations. Future studies can collect data from more time points across the course of adolescence to detect the associations examined in the current study with greater statistical power.

In addition, the findings are derived from a sample of Mexican immigrant families residing exclusively in Texas in the United States. Given the unique sociopolitical, economic, and cultural context of Texas—including its immigration policies, proximity to the U.S.-Mexico border, and regional community dynamics—caution is warranted when generalizing these results to Mexican immigrant populations in other U.S. regions or Mexico itself. Moreover, the study focused solely on Mexican immigrant families, precluding insights into whether the observed associations hold for other racial or ethnic groups. Future research can adopt national samples encompassing diverse geographic regions and racial and ethnic groups to elucidate similarities, differences, and contextual mechanisms underlying the studied phenomena across groups. Such efforts would strengthen the external validity of findings and inform culturally tailored policies and interventions.

Last, the current study was unable to reveal the mechanisms or the conditions underlying the associations examined. Future studies are needed to reveal the moderators (e.g., gender differences, mother-adolescent closeness, or discrepancies in mother-adolescent acculturation) or mediators (e.g., family functioning) of the associations between sociocultural stress and internalizing symptoms among mother-adolescent dyads at different adolescent ages.

## Conclusion

Mexican immigrant families navigate a complex sociocultural landscape marked by systemic inequities, cultural dissonance, and economic hardship, challenges that deeply impact maternal and child mental health, yet bidirectional stress spillover during sensitive developmental timing remains underexamined. This study advances the understanding of stress transmission dynamics in Mexican immigrant mother-adolescent dyads by identifying critical developmental periods when maternal or youth sociocultural stress most strongly influences the other’s internalizing symptoms. Contrary to hypotheses, maternal sociocultural stress (cultural misfit, foreigner stress, economic stress) showed minimal associations with adolescent internalizing symptoms across adolescence. However, adolescents’ sociocultural stress experiences, particularly cultural misfit and foreigner stress, were significantly linked to heightened maternal anxiety and depressive symptoms during early adolescence. Adolescents’ perceived economic stress was also related to heightened maternal anxiety in middle adolescence. These findings highlight the asymmetric nature of stress transmission in immigrant families, emphasizing adolescents’ role in shaping maternal mental health during sensitive developmental windows. By pinpointing early and middle adolescence as periods of heightened maternal vulnerability to youth stress, this study underscores the importance of developmental timing in family systems research and calls for targeted attention to adolescents’ sociocultural stressors in interventions aimed at improving family well-being.

## Supplementary information

Supplementary
