# Supplementary material for: Stress Spillover Among Mother-Adolescent Dyads in Mexican Immigrant Families: How It Varies from Early to Late Adolescence
Source: J Youth Adolesc. 2025 May 24;54(9):2339–53. doi: 10.1007/s10964-025-02197-6 (PMC12420714; doi:10.1007/s10964-025-02197-6)
Supplement: Supplementary file 1 — Supplementary [file 10964_2025_2197_MOESM1_ESM.docx]

**Stress Spillover among Mother-Adolescent Dyads in Mexican Immigrant Families: How It Varies from Early to Late Adolescence**

Wen Wen, Ashley Janyn Galvan, Ka I Ip, Yang Hou, Shanting Chen, Su Yeong Kim

**Supplementary**

| **Table S1** |  |  |  |  |  |  |  |  |  |  |  |
| --- | --- | --- | --- | --- | --- | --- | --- | --- | --- | --- | --- |
| *Longitudinal Measurement Invariance for Foreigner Stress* | | | | | | |  |  |  |  |  |
|  | *χ^2^* | *df* | *p* | CFI | RMSEA | *Δχ^2^* | *Δdf* | *p* | ΔCFI | ΔRMSEA |  |
| Mother |  |  |  |  |  |  |  |  |  |  |  |
| configural | 146.405 | 50.000 | <.001 | 0.930 | 0.057 |  |  |  |  |  |  |
| metric | 159.284 | 56.000 | <.001 | 0.926 | 0.055 | 12.879 | 6.000 | .045 | 0.004 | -0.002 |  |
| scalar | 172.372 | 62.000 | <.001 | 0.920 | 0.054 | 13.088 | 6.000 | .042 | 0.006 | -0.001 |  |
| Adolescent | |  |  |  |  |  |  |  |  |  |  |
| configural | 124.057 | 49.000 | <.001 | 0.953 | 0.050 |  |  |  |  |  |  |
| metric | 144.594 | 55.000 | <.001 | 0.944 | 0.052 | 20.537 | 6.000 | .002 | 0.009 | 0.002 |  |
| scalar | 198.005 | 61 | <.001 | 0.915 | 0.061 | 53.411 | 6.000 | <.001 | 0.029 | 0.009 |  |
| partial scalar | 156.594 | 59.000 | <.001 | 0.939 | 0.052 | 12.000 | 4.000 | .017 | 0.005 | 0.000 |  |
| *Note.* Metric invariance is established when the comparison between the configural and metric model, and scalar invariance is established when the comparison between metric and scalar models, each meets at least two out of the three following criteria (Widaman et al., 2010): Δχ^2^/Δ*df* is smaller than 5 given that Δχ^2^ is sensitive to sample size^2^ or Δχ^2^ is not significant (*p* >.05), ΔCFI < .01, and ΔRMSEA < .01. Both measures met scalar invariance. | | | | | | | | | | |  |
|  |  |  |  |  |  |  |  |  |  |  |  |
|  |  |  |  |  |  |  |  |  |  |  |  |
|  |  |  |  |  |  |  |  |  |  |  |  |
|  |  |  |  |  |  |  |  |  |  |  |  |

| **Table S2** |  |  |  |  |  |  |  |  |  |  |  |
| --- | --- | --- | --- | --- | --- | --- | --- | --- | --- | --- | --- |
| *Longitudinal Measurement Invariance for Cultural Misfit* | | | | | |  |  |  |  |  |  |
|  | *χ^2^* | *df* | *p* | CFI | RMSEA | *Δχ^2^* | *Δdf* | *p* | ΔCFI | ΔRMSEA |  |
| Mother |  |  |  |  |  |  |  |  |  |  |  |
| configural | 134.956 | 48.000 | <.001 | 0.942 | 0.055 |  |  |  |  |  |  |
| metric | 142.998 | 54.000 | <.001 | 0.941 | 0.052 | 8.042 | 6.000 | .235 | 0.001 | -0.003 |  |
| scalar | 149.631 | 60.000 | <.001 | 0.941 | 0.050 | 6.633 | 6.000 | .356 | 0.000 | -0.002 |  |
| Adolescent | |  |  |  |  |  |  |  |  |  |  |
| configural | 172.575 | 51.000 | <.001 | 0.929 | 0.063 |  |  |  |  |  |  |
| metric | 177.781 | 57.000 | <.001 | 0.929 | 0.059 | 5.206 | 6.000 | .518 | 0.000 | -0.004 |  |
| scalar | 189.690 | 63.000 | <.001 | 0.926 | 0.058 | 11.909 | 6.000 | .064 | 0.003 | -0.001 |  |
| *Note.* Metric invariance is established when the comparison between the configural and metric model, and scalar invariance is established when the comparison between metric and scalar models, each meets at least two out of the three following criteria (Widaman et al., 2010): Δχ^2^/Δ*df* is smaller than 5 given that Δχ^2^ is sensitive to sample size^2^ or Δχ^2^ is not significant (*p* >.05), ΔCFI < .01, and ΔRMSEA < .01. Both measures met scalar invariance. | | | | | | | | | | |  |
|  |  |  |  |  |  |  |  |  |  |  |  |
|  |  |  |  |  |  |  |  |  |  |  |  |
|  |  |  |  |  |  |  |  |  |  |  |  |
|  |  |  |  |  |  |  |  |  |  |  |  |

| **Table S3** |  |  |  |  |  |  |  |  |  |  |  |
| --- | --- | --- | --- | --- | --- | --- | --- | --- | --- | --- | --- |
| *Longitudinal Measurement Invariance for Depressive Symptoms* | | | | |  |  |  |  |  |  |  |
|  | *χ^2^* | *df* | *p* | CFI | RMSEA | *Δχ^2^* | *Δdf* | *p* | ΔCFI | ΔRMSEA |  |
| Mother |  |  |  |  |  |  |  |  |  |  |  |
| configural | 188.977 | 51.000 | <.001 | 0.938 | 0.067 |  |  |  |  |  |  |
| metric | 202.667 | 57.000 | <.001 | 0.934 | 0.065 | 13.690 | 6.000 | .033 | 0.004 | -0.002 |  |
| scalar | 218.418 | 63.000 | <.001 | 0.930 | 0.064 | 15.751 | 6.000 | .015 | 0.004 | -0.001 |  |
| Adolescent | |  |  |  |  |  |  |  |  |  |  |
| configural | 2353.938 | 1634 | <.001 | 0.92 | 0.027 |  |  |  |  |  |  |
| metric | 2426.734 | 1666 | <.001 | 0.915 | 0.027 | 72.796 | 32.000 | <.001 | 0.005 | 0.000 |  |
| scalar | 2527.113 | 1699 | <.001 | 0.908 | 0.028 | 100.379 | 33.000 | <.001 | 0.007 | 0.001 |  |
| *Note.* Metric invariance is established when the comparison between the configural and metric model, and scalar invariance is established when the comparison between metric and scalar models, each meets at least two out of the three following criteria (Widaman et al., 2010): Δχ^2^/Δ*df* is smaller than 5 given that Δχ^2^ is sensitive to sample size^2^ or Δχ^2^ is not significant (*p* >.05), ΔCFI < .01, and ΔRMSEA < .01. Both measures met scalar invariance. | | | | | | | | | | |  |
|  |  |  |  |  |  |  |  |  |  |  |  |
|  |  |  |  |  |  |  |  |  |  |  |  |
|  |  |  |  |  |  |  |  |  |  |  |  |
|  |  |  |  |  |  |  |  |  |  |  |  |

| **Table S4** |  |  |  |  |  |  |  |  |  |  |  |
| --- | --- | --- | --- | --- | --- | --- | --- | --- | --- | --- | --- |
| *Longitudinal Measurement Invariance for Anxiety* | | | | | |  |  |  |  |  |  |
|  | *χ^2^* | *df* | *p* | CFI | RMSEA | *Δχ^2^* | *Δdf* | *p* | ΔCFI | ΔRMSEA |  |
| Mother |  |  |  |  |  |  |  |  |  |  |  |
| configural | 164.073 | 51.000 | <.001 | 0.952 | 0.061 |  |  |  |  |  |  |
| metric | 169.796 | 57.000 | <.001 | 0.952 | 0.057 | 5.723 | 6.000 | .455 | 0.000 | -0.004 |  |
| scalar | 176.779 | 63.000 | <.001 | 0.951 | 0.055 | 6.983 | 6.000 | .322 | 0.001 | -0.002 |  |
| Adolescent | |  |  |  |  |  |  |  |  |  |  |
| configural | 165.118 | 51.000 | <.001 | 0.941 | 0.061 |  |  |  |  |  |  |
| metric | 185.661 | 57.000 | <.001 | 0.934 | 0.061 | 20.543 | 6.000 | .002 | 0.007 | 0.000 |  |
| scalar | 199.762 | 63.000 | <.001 | 0.929 | 0.060 | 14.101 | 6.000 | .029 | 0.005 | -0.001 |  |
| *Note.* Metric invariance is established when the comparison between the configural and metric model, and scalar invariance is established when the comparison between metric and scalar models, each meets at least two out of the three following criteria (Widaman et al., 2010): Δχ^2^/Δ*df* is smaller than 5 given that Δχ^2^ is sensitive to sample size^2^ or Δχ^2^ is not significant (*p* >.05), ΔCFI < .01, and ΔRMSEA < .01. Items within each subscale were averaged for mother’s model due to multicollinearity between items. Both measures met scalar invariance. | | | | | | | | | | |  |
|  |  |  |  |  |  |  |  |  |  |  |  |
|  |  |  |  |  |  |  |  |  |  |  |  |
|  |  |  |  |  |  |  |  |  |  |  |  |
|  |  |  |  |  |  |  |  |  |  |  |  |

**Figure S1**

*Distribution of Adolescent Ages across All Waves*

**
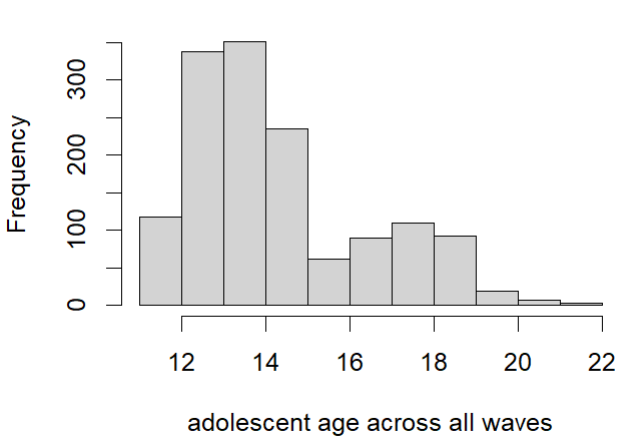
**

**Figure S2**

*Associations of Maternal Sociocultural Stress and Youth Internalizing Symptoms across Time (99% Confidence Interval)*


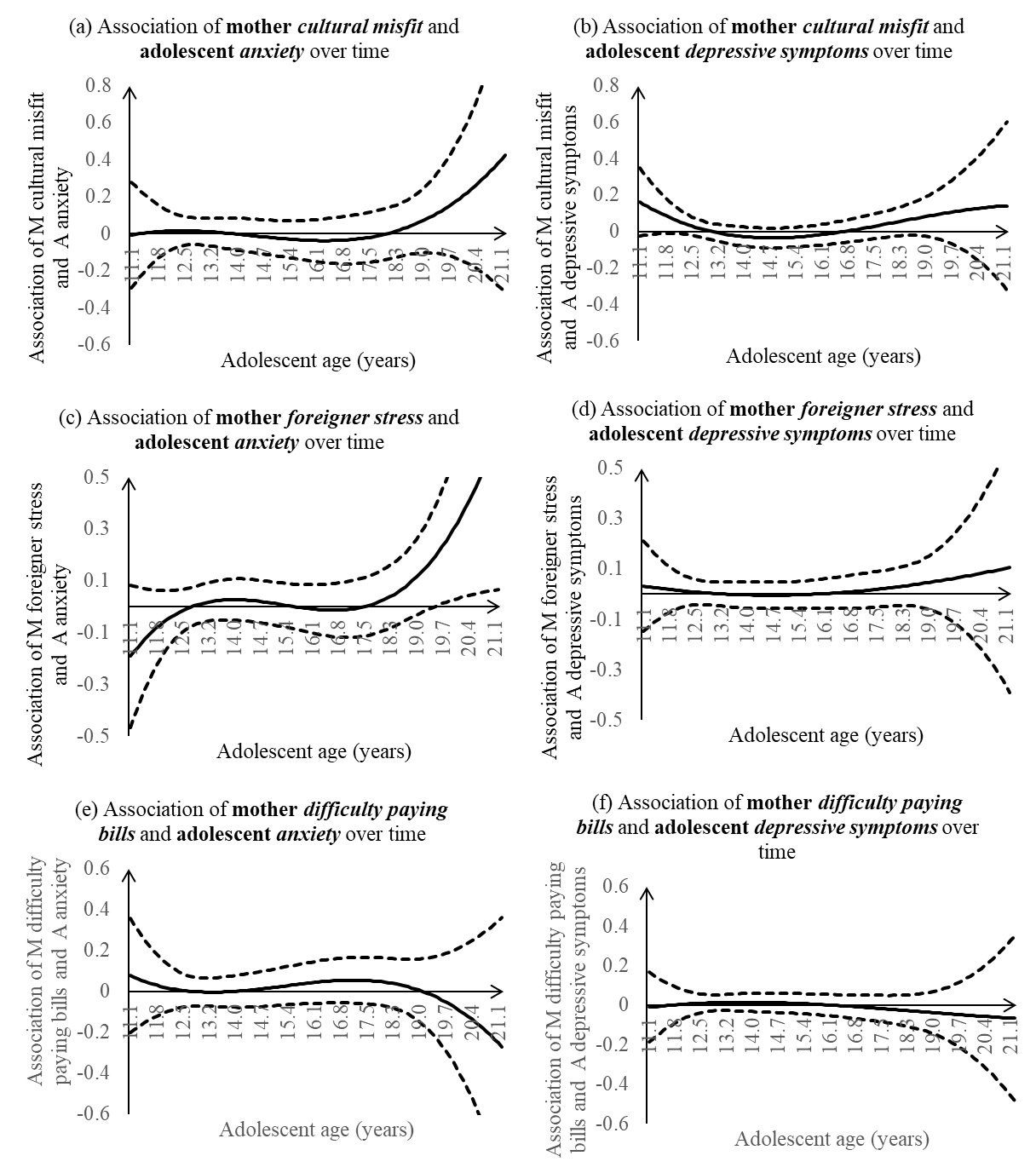


*Note.* Dashed lines represent point-wise 99% confidence intervals. A = adolescent, M = mother.

| **Figure S3** |
| --- |
| *Associations of Youth Sociocultural Stress and Maternal Internalizing Symptoms across Time*  *(99% Confidence Interval)* |


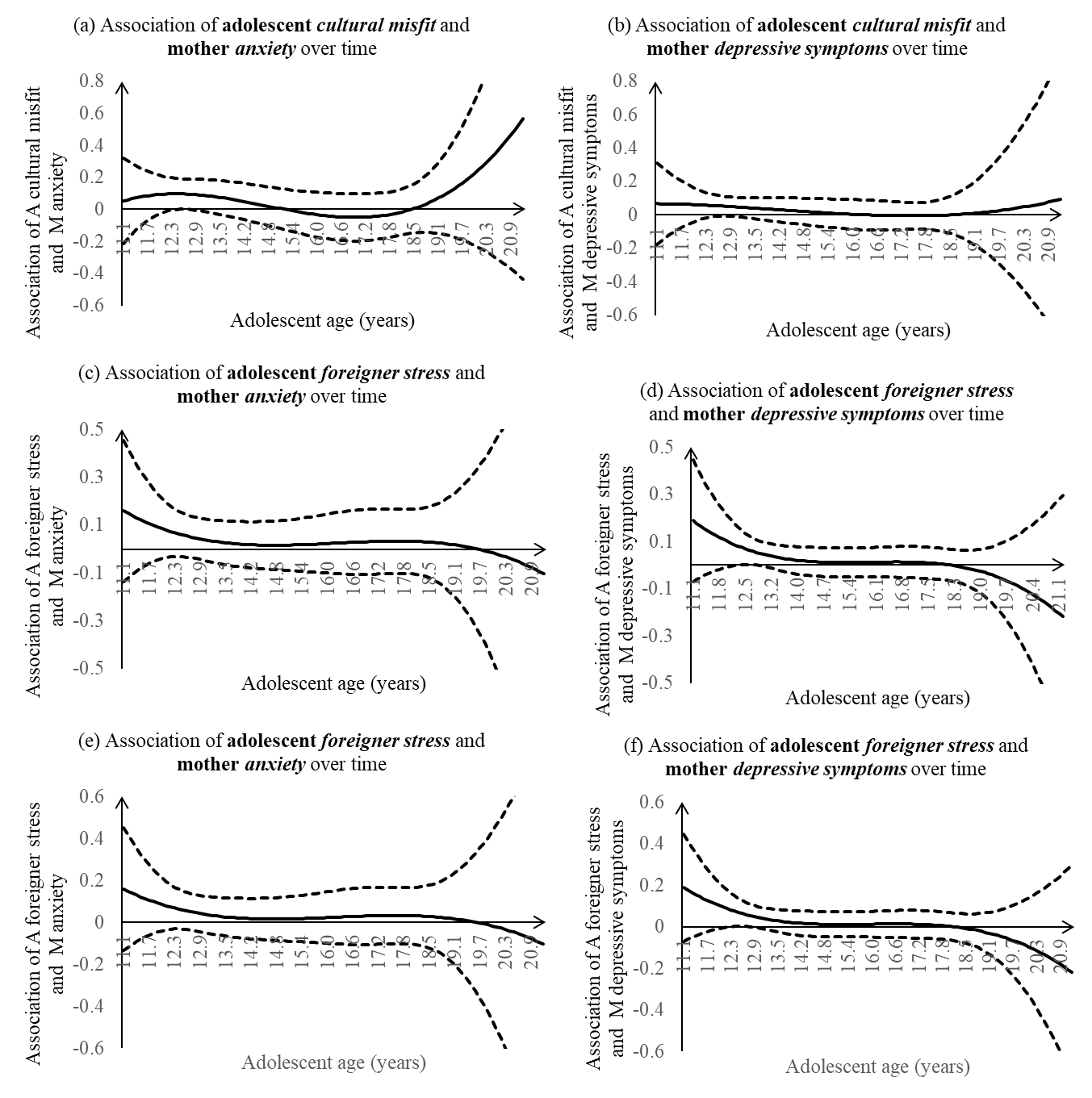


*Note.* Dashed lines represent point wise 99% confidence intervals. A = adolescent, M = mother.

**Scales**

*Cultural misfit*

How do you feel about living in the U.S.?

1. I feel that somehow I do not fit in with U.S Americans

2. I feel as though most U.S Americans do not understand

3. U.S Americans, on occasion, tell me that I am different from them

4. My ideas and opinions about important matters tend to differ from most people in this country

Responses:

1 Strongly disagree

2 Disagree

3 Neutral/Depends

4 Agree

5 Strongly agree

*Foreigner stress*

How much do you agree with the following statements?

1. Because of how I speak, people sometimes assume I am not a U.S American

2. When people look at me, they see a foreigner

3. People can notice an accent when I speak English

4. My opinions or ideas are not taken seriously because my English is not very good

Responses:

1 Strongly disagree

2 Disagree

3 Neutral/Depends

4 Agree

5 Strongly agree

*Depressive Symptoms*

Please choose the number for each statement that best describes how often you felt or behaved this way during the past week. During the past week...

1. I was bothered by things that I am usually not bothered by.

2. I did not feel like eating; my appetite was poor.

3. I could not shake off the blues (feeling down or bad) even with help from family or friends.

4. I felt just as good as any other person (other people).

5. I had trouble keeping my mind focused (pay attention) on what I was doing.

6. I felt depressed.

7. I felt everything was an effort (hard to do).

8. I felt hopeful (optimistic) about the future.

9. I thought my life had been a failure.

10. I felt fearful (scared) .

11. My sleep was restless (could not sleep well).

12. I was happy.

13. I talked less than usual.

14. I felt lonely.

15. I felt people were unfriendly.

16. I enjoyed life.

17. I had crying spells; I cried.

18. I felt sad.

19. I felt disliked by people.

20. I could not get going (get myself to do things).

Responses:

1. Rarely or none (less than 1 day)

2. Some or little of the time (1-2 days)

3. A lot of the time (3-4 days)

4. Most or all of the time (5-7 days)

*Anxiety*

Over the last 2 weeks, how often have you been bothered by the following problems?

1. Feeling nervous, anxious or on edge.

2. Worrying about what is going to happen.

3. Trouble relaxing.

4. Becoming easily annoyed or irritable.

Responses:

1. Not at all
2. Several days
3. More than half the days
4. Nearly every day

**Reference**

Widaman, K. F., Ferrer, E., & Conger, R. D. (2010). Factorial invariance within longitudinal structural equation models: Measuring the same construct across time. *Child Development Perspectives*, *4*(1), 10-18.
